# Noninvasive cross-sectional observation of three-dimensional cell sheet-tissue-fabrication by optical coherence tomography

**DOI:** 10.1016/j.bbrep.2015.05.001

**Published:** 2015-05-12

**Authors:** Yuji Haraguchi, Tatsuya Shimizu, Kiminori Mizuuchi, Hiroto Kawata, Mari Kobayashi, Yasushi Hirai, Shin-ichi Iwana

**Affiliations:** a Institute of Advanced Biomedical Engineering and Science, TWIns, Tokyo Women׳s Medical University, Tokyo 162-8666, Japan; b Panasonic Healthcare Co., Ltd., 2-38-5 Nishishinbashi, Minato-ku, Tokyo 105-8433, Japan; c Joint Graduate School of Tokyo Women׳s Medical University and Waseda University, 8-1 Kawada-cho, Shinjuku-ku, Tokyo 162-8666, Japan

**Keywords:** Cell adhesion, Cell sheet engineering, Noninvasive observation, Optical coherence tomography, Three-dimensional tissue fabrication

## Abstract

Cell sheet engineering allows investigators/clinicians to prepare cell-dense three-dimensional (3-D) tissues, and various clinical trials with these fabricated tissues have already been performed for regenerating damaged tissues. Cell sheets are easily manipulated and 3-D tissues can be rapidly fabricated by layering the cell sheets. This study used optical coherence tomography (OCT) to noninvasively analyze the following processes: (1) adhesions between layered cell sheets, and (2) the beating and functional interaction of cardiac cell sheet-tissues for fabricating functional thicker 3-D tissues. The tight adhesions and functional couplings between layered cell sheets could be observed cross-sectionally and in real time. Importantly, the noninvasive and cross-sectional analyses of OCT make possible to fabricate 3-D tissues by confirming the adherence and functional couplings between layered cell sheets. OCT technology would contribute to cell sheet engineering and regenerative medicine.

## Highlights

• Development of an OCT system to noninvasively analyze three-dimensional cell sheet-tissues.
• Noninvasive and cross-sectional observation of the adhesions between layered cell sheets in detail.
• Noninvasive and cross-sectional observation of beating three-dimensional cardiac tissues.

## 1 Introduction

Damaged tissues have already been clinically treated with a variety of regenerative therapies using functional cells and bioengineered tissues [1], [2]. We have proposed scaffold-free tissue engineering, called “cell sheet engineering”, utilizing temperature-responsive culture dishes, which possess reversible hydrophilic/hydrophobic properties that are simply controlled by culture temperature [3]. Cell sheets are comprised of only cells and a biological extracellular matrix (ECM), which means that cell-dense three-dimensional (3-D) tissue can be fabricated by simply layering cell sheets without any need for scaffolds [4], [5], [6], [7]. Three-dimensional cell sheet-tissues have been applied to regenerate damaged tissues, and cell sheet-therapy has already been used clinically in six different fields [8], [9], [10], [11], [12], [13], [14], [15], [16], [17]. The presence of ECM is thought to promote the tight, rapid attachment between individual layered cell sheets [9], [18]. There have been very few studies that could observe cross-sections of cell sheet-tissues noninvasively to analyze adhesion and functional communications due to the technical difficulty [19], [20].

Three-dimensional tissues and their microstructures can be observed in cross-section by optical coherence tomography (OCT) in real time [21]. The technology has been applied in several clinical fields, where the safety, non-invasiveness and feasibility have been confirmed; at present, OCT had become an important method in clinical examination [22], [23], [24], [25], [26], [27], [28].

Recently, we have developed an OCT system, which can observe living cell sheets in cross-section [29]. In this study, the in-vitro fabrication of 3-D tissues with cell sheet engineering, as well as the beating and functional coupling of 3-D cardiac tissues were analyzed noninvasively by OCT. This technology could be an invaluable method in the fields of cell sheet engineering, tissue engineering, and regenerative medicine.

## 2 Materials and methods

All animal experiments were performed in accordance with the experimental procedures approved by the Committee for Animal Research of Tokyo Women׳s Medical University.

### 2.1 Cell culture and cell sheet preparation

C2C12 murine skeletal myoblast lines (Sumitomo Dainippon Pharma, Osaka, Japan) and NIH3T3 murine embryonic skin fibroblast lines (ATCC^®^ CRL-1658^™^) [30] were used in this study. After C2C12 or NIH3T3 cells were mixed with a medium [Dulbecco׳s modified Eagle׳s medium (Sigma-Aldrich, St. Louis, MO, USA) supplemented with 10% fetal bovine serum (Japan Bio Serum, Nagoya, Japan) and 1% penicillin-streptomycin (Invitrogen Life Technologies, CA, USA)], 6.0×10^5^ cells were seeded onto a 35-mm temperature-responsive culture dish (UpCell^®^ dish) (CellSeed, Tokyo, Japan) and then cultured for 3 days at 37 °C. Rat neonatal cardiac cell sheets were fabricated using UpCell^®^ dishes according to a previous report [7]. To harvest these cell sheets, the culture dishes were placed in a separate CO_2_ incubator set at 20 °C. To fabricate 3-D tissues, cell sheets were layered on a 35 mm polystyrene culture dish (Corning, NY, USA) by pipetting as described in previous reports [6], [7].

### 2.2 Optical coherence tomography (OCT)

Recently, an OCT system to analyze cell sheets has been established [29]. The adhesions between a cell sheet and a polystyrene culture dish, and between layered cell sheets, were observed at 37 °C. The location of spaces, which was characterized by the intensity of the OCT signal, between (1) a cell sheet and the dish, or (2) layered cell sheets is displayed as red in the image. The vertical resolution of the OCT was approximately 9 μm, and the horizontal resolution was approximately 20 μm. The beatings of cardiac cell sheets were observed and analyzed at 32 fps (frames per second). Within cardiac cell sheets, areas determined as beating within the cell sheet were those places where the correlation of OCT signals at intervals of 90 ms was lower than a predetermined level. The beating areas were marked with green colored markers.

## 3 Results and discussion

### 3.1 Observation of C2C12 and NIH3T3 cell sheets by OCT

**Fig. 1:**
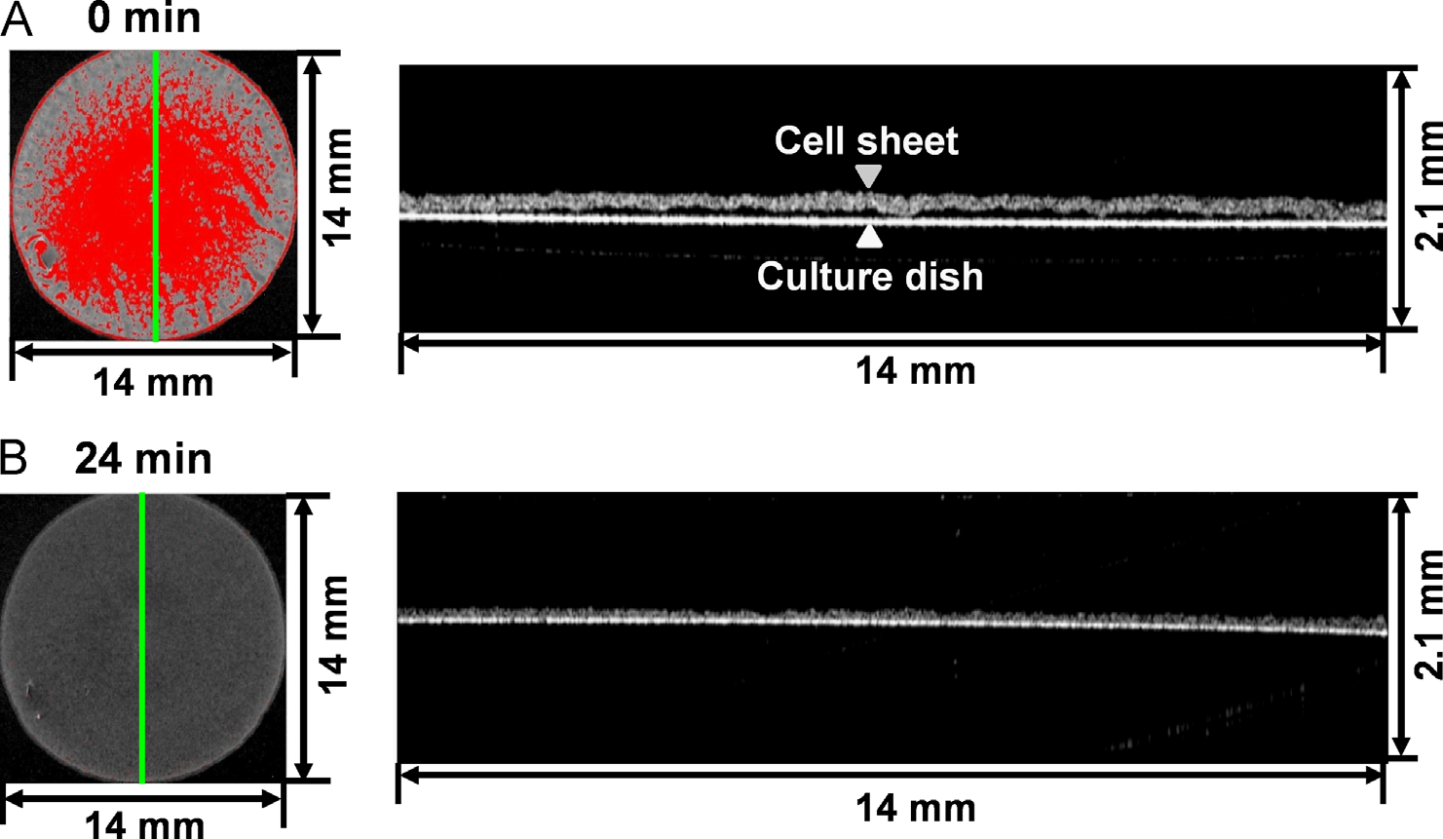
Observation of an NIH3T3 cell sheet onto a polystyrene culture dish by OCT. Upper and lower left pictures are the merged images of (1) the top-view observations of an NIH3T3 cell sheet onto the dish at 0 (A) and 24 min (B) after transfer, respectively; and (2) the red-colored space images between the cell sheet and the dish, and the right panels are cross-sectional observations of the cell sheet. Green lines in the left pictures show the cutting sites in the right panels.

A cell sheet with culture medium was transferred onto a polystyrene culture surface as described in previous reports [4], [5], [6], [7]. After cell sheets were transferred onto the culture dish, medium was removed to facilitate spreading of the cell sheet. After a short-term cultivation of less than 30 min, the spaces were found to decrease rapidly, and when no spaces were observed, the transferred cell sheet was found to be in a smooth plane form (Fig. 1B). The rapid time-course decrease in spaces between the cell sheet and the dish was clearly recorded (Video 1). When new medium was added to the cell sheet to prevent drying out, the cell sheet continued to adhere onto the dish (Video 1), showing a tight attachment between the cell sheet and the dish. Similar adhesion processes were also observed in C2C12 cell sheets onto the dish using OCT, and tight attachment between the cell sheet and the dish was confirmed (data not shown). We have been attempting to develop a cell culture surface with higher functionality that is able to precisely control the attachment/detachment of cells by modulating the chemical structure of the surface; for example, hydrophilically modified cell culture surfaces can accelerate cell sheet detachment [31], [32], [33]. At present, surfaces that have been developed are mainly evaluated by top-view photography. Because OCT allows us to analyze the attachment/detachment of cell sheets cross-sectionally and noninvasively, the technology will use as an optimal system to assess and quantify cell culture surfaces that accelerate the attachment/detachment of cell sheets.

Supplementary material related to this article can be found online at doi:10.1016/j.bbrep.2015.05.001.

The following is the Supplementary material related to this article Video 1.

Video 1 Time-course observation of the adherence process of an NIH3T3 cell sheet onto a polystyrene culture surface by OCT. The upper movie and the lower movie show a merged image of the top view and red-color space image, and the cross-sectional observation of the cell sheet-adhesive, respectively. The movies show the phenomenon at a speed 60 times faster than real time. The green line in the lower movie shows the cross-sectional surface of the upper movie. The numbers on the right are the incubation times (_min_) after the transfer of a cell sheet.

**Fig. 2:**
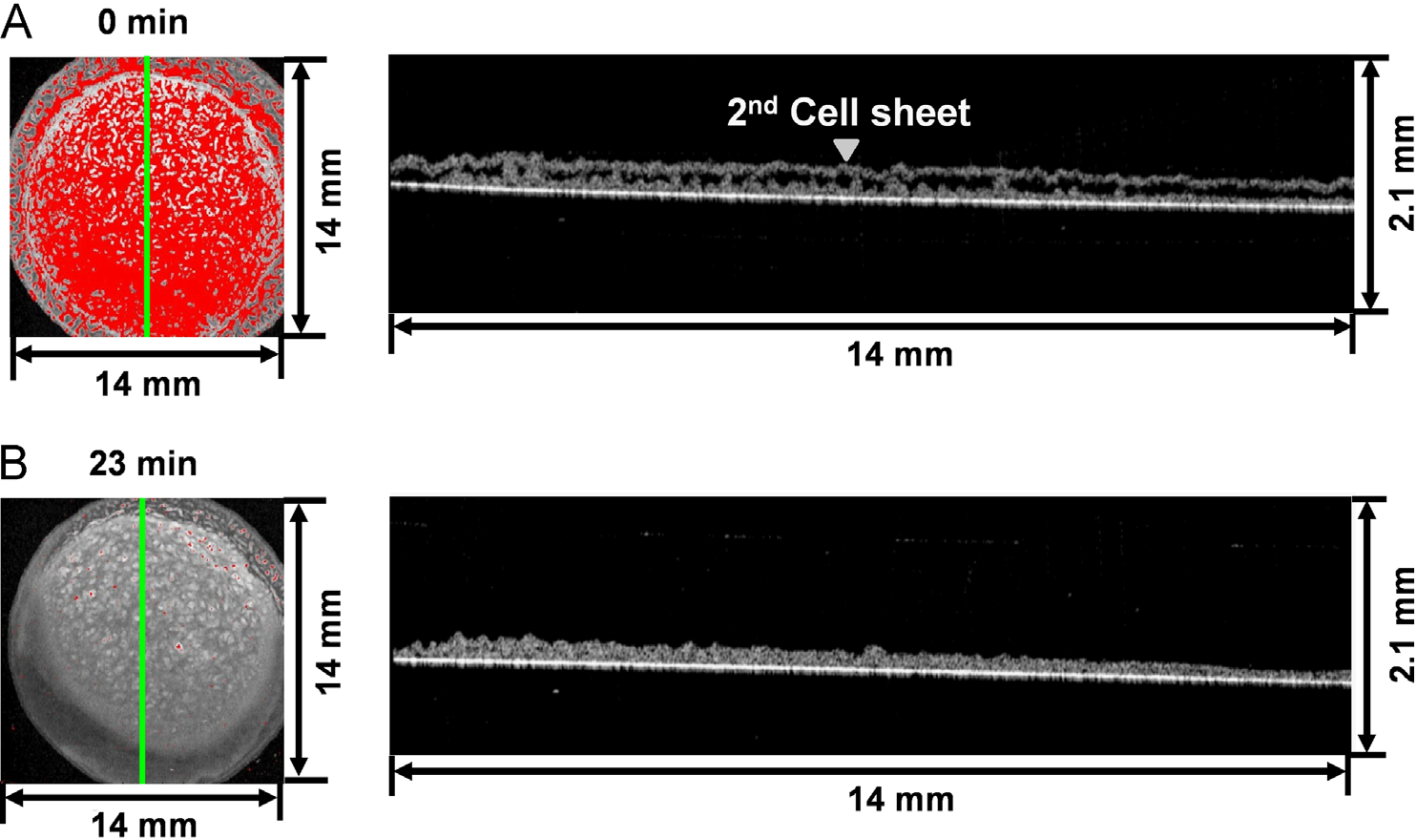
Observation of two C2C12 cell sheets by OCT. Upper and lower left pictures are merged images of (1) the top-view observations of two C2C12 cell sheets at 0 (A) and 23 min (B) after layering, respectively; and (2) the red-colored space images between the cell sheets, and the right panels are the cross-sectional observations of the cell sheet. Green lines in the left pictures show the cutting sites in the right panels.

For observing and analyzing the cross section and time course of the adhesion between layered cell sheets, cell sheets were layered. After the first C2C12 cell sheet was adhered onto a culture surface, another C2C12 sheet was layered onto the first cell sheet, and medium was removed to facilitate spreading the second cell sheet. After layering, the time course showed a rapid decrease in spaces between the two cell sheets was clearly observed within 30 min by OCT (Fig. 2 and Video 2). When new medium was added to the cell sheet to prevent drying out, the two cell sheets continued to adhere (Video 2), indicating that there was a tight attachment between the cell sheets. These results show that OCT can detect the adhesion between layered cell sheets, as well as between a cell sheet and the culture surface. Therefore, OCT technology will be also used in an assessment system to quantify the search for culture methods, which accelerate the attachment between multi-layered cell sheets biologically and physically.

Supplementary material related to this article can be found online at doi:10.1016/j.bbrep.2015.05.001.

The following is the Supplementary material related to this article Video 2.

Video 2 Time-course observation of the adhesion process between double-layered C2C12 cell sheets by OCT. The upper movie and the lower movie show a merge image of the top view and the red-color space image, and the cross-sectional observation of the cell-sheet-adhesive, respectively. The movies show the phenomenon at a speed 60 times faster than real time. The green line in the lower right movie shows the cross-sectional surface of the upper movie. The numbers on the right are incubation times (_min_) after cell sheet layering.

**Fig. 3:**
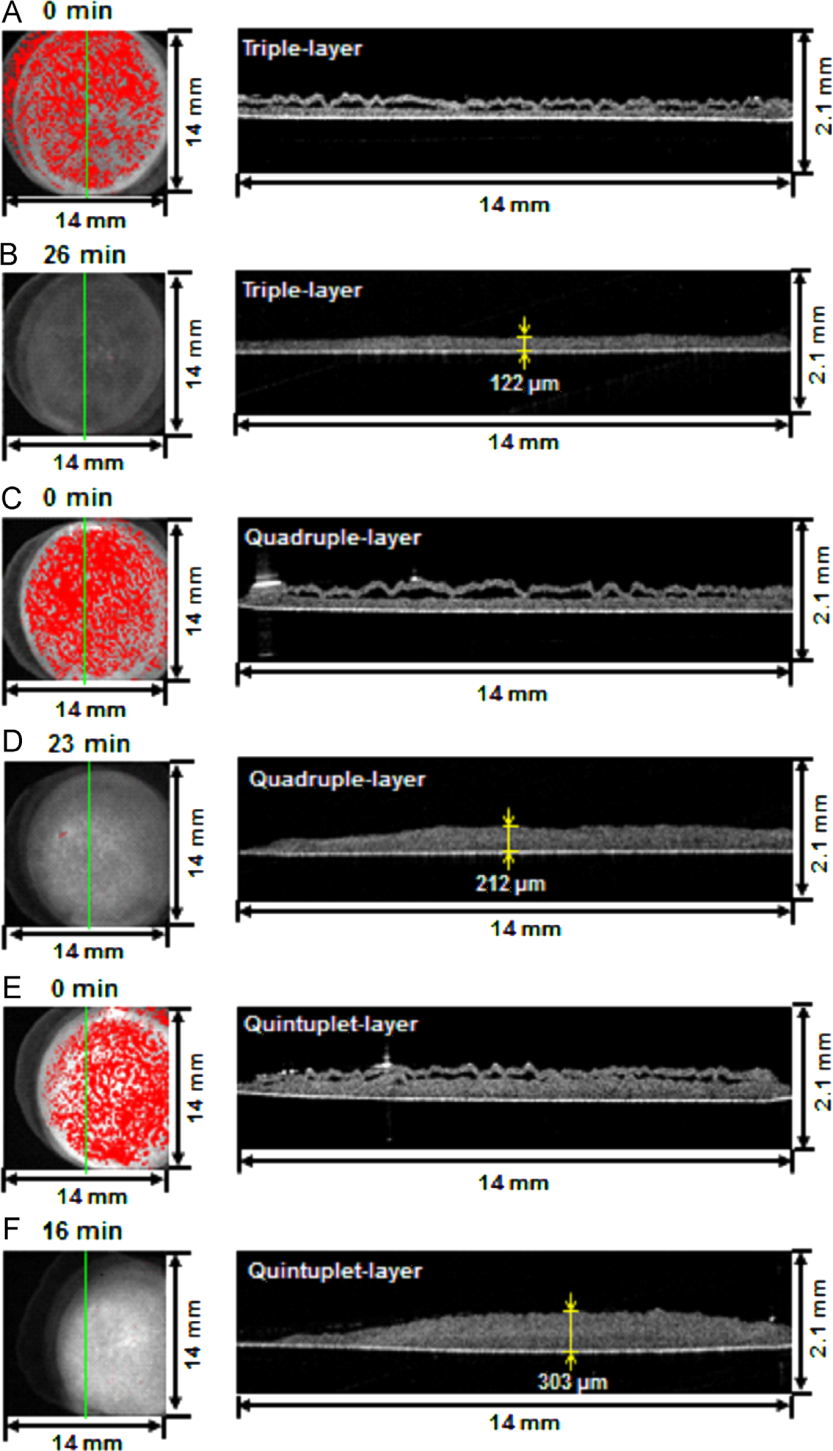
Observations of multi-layered C2C12 cell sheets by OCT. Three of the pictures on the left show the merged images of (1) the top-view of a triple-layered (A), quadruple-layered (C), and quintuple-layered (E) C2C12 cell sheets just after layering, and (2) the red-colored space images between layered cell sheets. The three other pictures on the left show the merged images of (1) the top-view of a triple-layered (B), quadruple-layered (D), and quintuple-layered (F) C2C12 cell sheets at each time after layering (B: 26 min; D: 23 min; F: 16 min), and (2) the red-colored space images between layered cell sheets. The panels on the right show these same cross-sectional observations. Thicknesses shown in the three photographs (B, D, F) were calculated at the points indicated. Green lines in the left pictures show the cutting sites in the right panels.

C2C12 cell sheets were stacked to form a multi-cell layer tissue while simultaneously observing their cross section by OCT. After layering two C2C12 cell sheets, a third cell sheet was stacked onto the double-layer cell sheet. Just after layering, spaces between the third cell sheet and the double-layer cell sheet could be observed clearly (Fig. 3A), then after a short incubation of less than 30 min, complete attachment between the cell sheets was observed (Fig. 3B). Using a similar procedure, harvested cell sheets were successfully stacked up into quintuple-layer tissue, and the same adhesion processes between multi-layered cell sheets was clearly detected (Fig. 3). Only a few spaces between the cell-sheet layers were detected, showing tight adhesions between the multi-layered cell sheet constructs. When cell sheets have been layered to fabricate 3-D tissues, whether to adhere a cell sheet to the culture surface, or between layered cell sheets, medium should be removed, and after a suitable incubation time, new medium is added to avoid the drying out [4], [5], [6], [7]. This manipulation has been performed experientially, and its feasibility and efficacy were confirmed by OCT. In addition, the incubation time between removing the medium and adding new medium is largely dependent on the experience of the investigators. Importantly, OCT allows investigators and clinicians to manipulate cell sheets while confirming noninvasively the adhesion between a cell sheet and the culture surface, or between layered cell sheets in a 3-D tissue-fabrication. Furthermore, the thicknesses at arbitrary points in the cell sheet-tissues could be easily measured, as shown in Fig. 3.

### 3.2 Observation of rat cardiac cell sheets by OCT

**Fig. 4:**
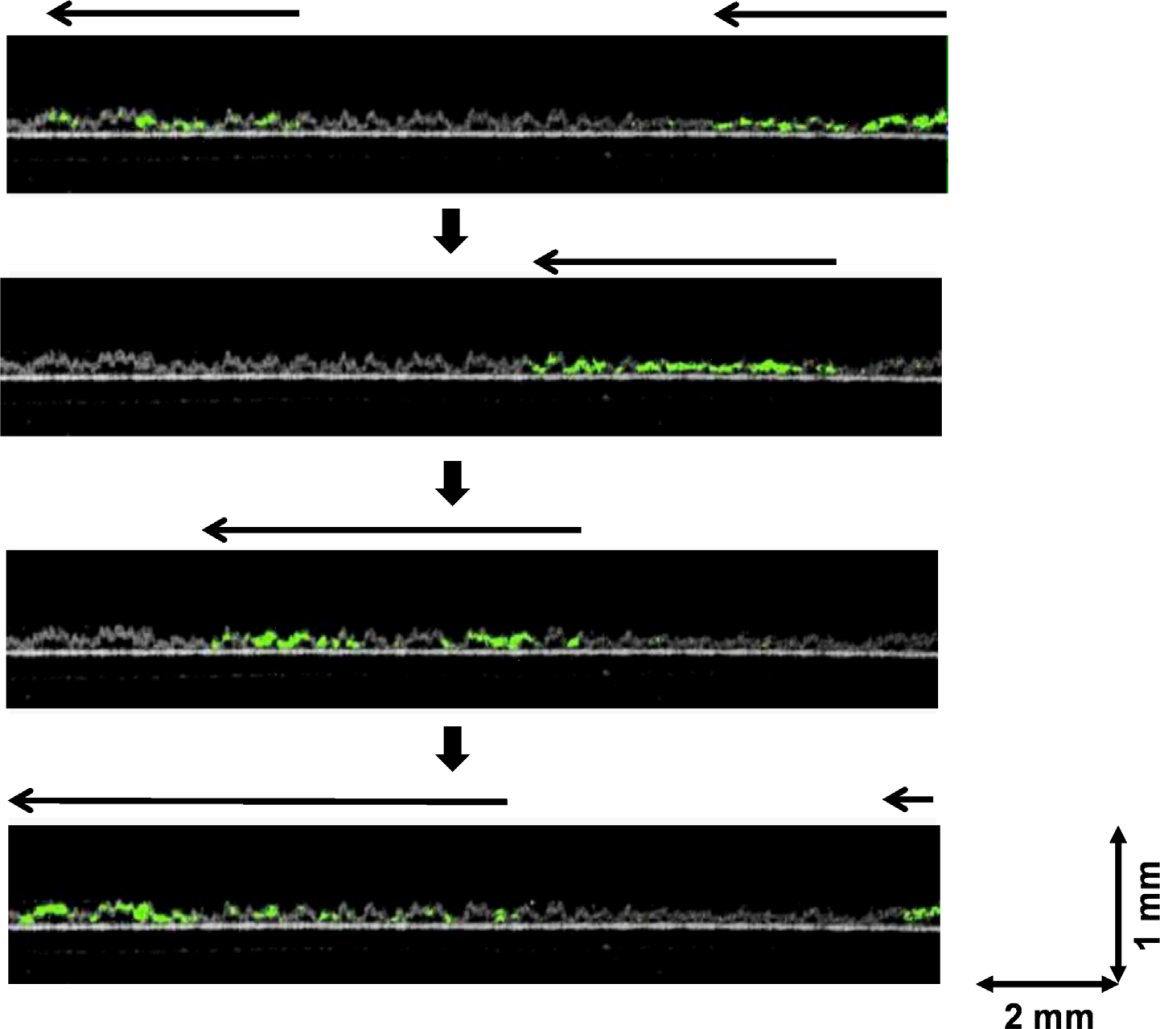
Observation of the beating of a rat cardiac cell sheet by OCT. Beating areas within the cell sheet just after transfer onto a polystyrene culture dish are indicated with green colored markers, at which the correlation of the OCT signals for a short interval (90 ms) was lower than the predetermined level. The transmission of beating areas (arrows) is shown clearly.

A rat cardiac cell sheet detaching from a temperature-responsive culture dish was cross-sectionally analyzed. The cell sheet was detached from both edges, and the detachment was recorded in movie data by OCT (Video 3), which shows the detaching of a beating cell sheet. The beating of a detached cardiac cell sheet was analyzed by OCT. Within the cell sheet, beating areas were marked with green colored markers, where the correlation of OCT signals for short intervals (90 ms) was lower than the predetermined level. Fig. 4 and Video 4 clearly show the transmission of green areas, namely, the beating areas within the cell sheet, which indicate that the transmission of action potentials within a cardiac cell sheet could be noninvasively detected in cross-section by OCT. Next, harvested cardiac cell sheets then were layered to form a double-layer tissue while observing it by OCT, which showed that the cell sheets seemed to beat synchronously (Video 5). This data showed that the 3-D transmission of beating cardiac cells within a multi-layered cell sheet could be observed by OCT, and suggests a functional coupling between layered cell sheets, confirming the previous observations in our laboratory [4], [7], [19]. The noninvasive observation will also contribute to the electrophysiology of cardiac tissues. At present, we are preparing the investigation of the electrical and functional coupling processes between layered cardiac cell sheets in detail using the technique of the combination of OCT system and a multiple-electrode extracellular recording system.

Supplementary material related to this article can be found online at doi:10.1016/j.bbrep.2015.05.001.

The following is the Supplementary material related to this article Video 3, Video 4, Video 5.

Video 3 Observation of a rat cardiac cell sheet detaching from a temperature-responsive culture surface by OCT. The movie shows that a rat cardiac cell sheet is detaching while beating as the temperature decreases. Although the movie shows the phenomenon at real time speed, there are two time-gaps, which are shown as a “Time-gap” in the Video.

Video 4 Observation of the beating of a rat cardiac cell sheet by OCT. The movie shows cross-sectionally the beating of a rat cardiac cell sheet. Beating areas within the cell sheet just after transfer onto a polystyrene culture dish are shown with green colored markers, at which the correlation of the OCT signals for a short interval (90 ms) was lower than the predetermined level. The movie shows the phenomenon at real time speed.

Video 5 Observation of the beating of double-layered rat cardiac cell sheets by OCT. The movie shows the cross-sectional observation of double-layered rat cardiac cell sheets. Beating areas within the cell sheet are shown with green colored markers. The movie shows the phenomenon at real time speed. Beatings appear to be transmitted from the area around the arrow.

Clinical trials of autologous skeletal myoblast-sheet transplantation into heart disease patients are now underway. The first patient, who suffered from a dilated cardiomyopathy has received autologous cell sheet therapy, and is now in good clinical condition [12]. In the first clinical therapy, quadruple-layered myoblast sheets were used. It is generally accepted that enormous numbers of cells (10^9^ cell-level per patient) are necessary for treating conditions such as cardiovascular disease and diabetes [34], and layered cell sheets make it possible to transplant enormous numbers of cells onto target tissues. OCT observation then allowed us to detect tight and complete adhesions between multi-layered cell sheets noninvasively (Fig. 2, Fig. 3). OCT can be a powerful tool for analyzing the quality of engineered tissues in clinical cell sheet-therapy.

While the therapeutic effects of adult stem/progenitor cells including skeletal myoblasts are generally thought to be mainly due to the paracrine effects of various factors secreted from implanted cells [35], beating cardiac cells and cardiac tissue are expected to contribute to the mechanical support of damaged heart tissue via electrical and functional couplings as well as their paracrine effects [36], [37]. In an effort to make a more advanced regenerative therapy, attempts at engineering beating myocardial tissue using cardiac cell sheets have already been performed [4]. In fact, multi-layered cardiac cell sheets give good therapeutic effects in rat models [38], [39]. In addition, we have also succeeded in the fabrication of spontaneously beating human cardiac cell sheets, using human induced pluripotent stem cells (hiPSCs) [20], [40]. hiPSC-derived cardiac cell sheets have been shown to be feasible and safe in a large animal model [41]. In the near future, cardiac cell sheets will be used clinically for regenerating damaged heart tissues. This study showed that beating of cardiac cell sheet-tissues could be cross-sectionally detected by OCT (Movies 4 and 5). OCT could be also be used to evaluate the beating and functional coupling of engineered cardiac tissues.

Recently, we observed cell-sheet-transfer-process and the adhesion between a cell sheet and target tissue by OCT using the rat model [29]. At present, to evaluate the potential for clinical use, we are attempting to observe and analyze the transplantation of cell sheets onto beating heart tissue using a porcine model by OCT. OCT could also be used clinically, as a method to observe and analyze adhesions and functional couplings between transplanted cell sheets and target tissues, as well as to assess the quality of engineered tissues before transplantation.

In this study, the dynamics of layered cell sheets were observed noninvasively by OCT. OCT technology allowed us to analyze in real time the cross-sectional adhesion between (1) multi-layered cell sheets, and (2) beating 3-D cardiac tissues in detail. Using this method, we were able to confirm the rapid adhesion and functional coupling of 3-D tissues. In addition, OCT observation could allow investigators and clinicians to fabricate three-dimensional tissues by confirming the adherence and functional coupling between layered cell sheets. We are confident that OCT technology can be used a powerful method in cell sheet engineering and the clinical application.
