# Supplementary material for: Noninvasive cross-sectional observation of three-dimensional cell sheet-tissue-fabrication by optical coherence tomography
Source: Biochem Biophys Rep. 2015 May 12;2:57–62. doi: 10.1016/j.bbrep.2015.05.001 (PMC5668641; doi:10.1016/j.bbrep.2015.05.001)
Supplement: Supplementary file 6 — Transparency document [file mmc6.doc]

**Conflict of Interest**

Yuji Haraguchi has no conflicts of interest to declare. Tatsuya Shimizu is a stakeholder of CellSeed Inc. Kiminori Mizuuchi, Hiroto Kawata, Mari Kobayashi, Yasushi Hirai, and Shin-ichi Iwana are employees of Panasonic Healthcare Co., Ltd. Tokyo Women’s Medical University was receiving research funds from CellSeed Inc., and Panasonic Healthcare Co., Ltd.
